# Assessing Community and Social Media Influence to Increase Influenza Vaccine Uptake among Youth in Soweto, South Africa (The Bambisana Study): Protocol for a Mixed Methods Pretest-Posttest Intervention Study

**DOI:** 10.2196/60481

**Published:** 2025-06-17

**Authors:** Janan Dietrich, Catherine Hill, Gugulethu Tshabalala, Tshepiso Msibi, Stefanie Vermaak, Mulalo Mashamba, Nellie Myburgh, Sarah Malycha, Isabella Goldstein, Elliot Grainger, Prima Alam, Kimberley Gutu, Kennedy Otwombe, Heidi J. Larson, Ziyaad Dangor

**Affiliations:** 1 African Social Sciences Unit of Research and Evaluation (ASSURE) Faculty of Health Sciences University of the Witwatersrand Johannesburg South Africa; 2 Health Systems Research Unit South African Medical Research Council Cape Town South Africa; 3 South African Medical Research Council Vaccines and Infectious Diseases Analytics Research Unit Faculty of Health Sciences University of the Witwatersrand Johannesburg South Africa; 4 Perinatal HIV Research Unit (PHRU) Faculty of Health Sciences, School of Clinical Medicine University of the Witwatersrand Johannesburg South Africa; 5 Department of Social Science Africa Health Research Institute Mtubatuba South Africa; 6 Department of Infectious Disease Epidemiology and Dynamics, Vaccine Confidence Project Faculty of Epidemiology and Population Health London School of Hygiene & Tropical Medicine London United Kingdom; 7 School of Public Health Faculty of Health Sciences University of the Witwatersrand Johannesburg South Africa; 8 Institute for Health Metrics and Evaluation University of Washington Seattle United States

**Keywords:** influenza, vaccines, vaccine confidence, youth, South Africa, social media

## Abstract

**Background:**

Seasonal influenza has an estimated global reach of 3-5 million infections, with 290,000-650,000 influenza-related deaths yearly. Despite its efficacy in reducing morbidity and mortality, influenza vaccination rates remain low globally and in South Africa. Youth between the ages of 18 and 34 years are not prioritized for influenza vaccines although influenza surveillance in South Africa shows that individuals aged 19-44 years present the highest asymptomatic episodes and the lowest medically attended illness. This creates an opportunity to investigate if and how vaccine demand can be created in the absence of clear imperatives to vaccinate. The study tests the effectiveness of tailored, context-specific education, and community engagement, including community and social media to increase influenza vaccination uptake. Tailored, context-specific education, community engagement, reliable vaccine supply, and free, localized access are all critical for improving perceptions of, increasing confidence in, and motivating the uptake of vaccination.

**Objective:**

This study will explore strategies to increase influenza vaccine uptake amongst economically marginalized youth aged 18-34 years in Soweto (South-Western Townships), South Africa, where influenza vaccines are not universally accessible through the public health system for this age group.

**Methods:**

The Bambisana Study uses an innovative approach, including community influencers and social media to increase the uptake of influenza vaccines through designing and testing an integrated communications strategy targeted at economically marginalized youth in Soweto, South Africa. The study uses a mixed methods pretest-posttest intervention design to test the effects of the interventions. The intervention will consist of the following components: (1) social media campaign, (2) microinfluencers on and offline, and (3) nonsocial media focused, offline microinfluencer-led engagement within communities. Quantitative data will be collected using a randomized household sample pre- and posttests, and clinic surveys with vaccinees and clinic attendees who declined vaccination. Focus group discussions (FGDs) will be conducted pre- and post intervention with participants aged ≥18 years, and 20 key informant interviews (KIIs) will be conducted with key influencers including religious leaders, traditional healers, and youth leaders. FGDs and KIIs will be audio-recorded and transcribed into English for analysis using framework thematic analysis, and quantitative data analyses will be conducted using SAS Enterprise (Guide 7.15; SAS Institute).

**Results:**

This study was funded in December 2022, with recruitment having started in May 2023. As of May 2024, all data collection is complete, with data analyses and preparation of peer-reviewed publications in progress. The first results are expected to be submitted for publication in November 2024.

**Conclusions:**

Enhancing perceptions of, bolstering confidence in, and fostering uptake of vaccination relies heavily on the efficacy of yearly influenza vaccination initiatives, personalized education tailored to specific contexts, active community involvement, consistent vaccine availability, and easily accessible, cost-free distribution channels at the local level.

**International Registered Report Identifier (IRRID):**

DERR1-10.2196/60481

## Introduction

Globally, seasonal influenza causes an estimated 3-5 million infections, and 290,000-650,000 deaths annually [1,2]. Estimates for the influenza burden of disease are limited in Africa; however, hospitalization and mortality are higher in low- and middle-income countries (LMICs) than in high-income countries [3,4]. In South Africa, an estimated 6,000-11,000 people die from influenza every year, with approximately half of these deaths in older individuals, and people living with HIV [5]. Seasonal outbreaks of influenza pose challenges to the health care system, leading to increased hospitalizations, outpatient visits, and strain on health care resources [6]. Vulnerable populations, including the older individuals, young children, pregnant women, and individuals with underlying medical conditions are at the highest risk of severe infection [7].

While nonpharmaceutical interventions can play a role in decreasing influenza infections, vaccination is deemed pragmatic and endorsed by the World Health Organization (WHO) as the recommended method for preventing seasonal influenza [1]. Overall, vaccine efficacy to prevent influenza infection ranges from 40% to 60%, with higher vaccine efficacy (82%) in reducing severe illness and hospitalization [8,9]. Therefore, the influenza vaccine is especially recommended for those who have chronic illnesses, those who are pregnant, and older individuals [10-12]. Influenza vaccination campaigns typically prioritize these groups during the influenza vaccination season [7,13]. However, an investigation into the seasonal influenza burden in urban and rural areas in South Africa showed that children younger than 12 years and those aged 19 and 44 years have an elevated risk of acquiring seasonal influenza [14]. Whilst severe disease is less likely, vaccinating younger adults has the added indirect benefit of providing herd immunity to the family and communities [15,16]. Furthermore, vaccination offers potential economic benefits related to the out-of-pocket expenses incurred by patients to treat influenza-related symptoms. An estimated annual cost of US $111.3 million is incurred by the government, while out-of-pocket costs incurred by the patient were reported to be US $40.7 million [17]. Individuals are at risk of financial loss due to absence from work and missed opportunities for those who are self-employed [18]. Despite recommendations, seasonal influenza vaccine uptake in the most vulnerable groups is low in LMICs, including South Africa [19]. Accessibility, lack of knowledge, and risk perception have been cited as the most significant barriers to vaccination in South Africa [20].

Despite the demonstrated effectiveness of vaccines in significantly reducing morbidity and mortality of affected individuals, youth are less proactive in health care–seeking behaviors due to low-risk perception and are not necessarily encouraged to vaccinate for influenza and other infectious diseases [9,21-23]. For COVID-19, youth were not initially prioritized for vaccination rollout, yet they might have been in contact with vulnerable populations [24,25]. In addition, evidence suggests that low vaccination rates are in part due to low health care–seeking behaviors among youth. Health care–seeking behaviors are the actions involved in achieving and maintaining a healthy condition [26,27]. Various factors, both individual and related to health care providers, can influence young people’s health care–seeking behaviors [28].

Targeted health promotion interventions can increase health care–seeking behaviors and uptake of vaccination [29]. Influenza surveillance shows that individuals aged 19-44 years present the highest asymptomatic episodes and the lowest medically attended illness [14,30]. Studies undertaken in sub-Saharan Africa have shown that leveraging community involvement in immunization programs positively contributes to vaccine confidence and uptake [31,32]. In the case of HIV, for example, awareness campaigns to lower the burden of HIV and AIDS epidemics in Southern African countries have proven vital in reducing the rise of the disease [33]. The use of television series to promote positive sexual health and prevent HIV effectively engaged the youth by challenging old models of education [34]. The use of social media campaigns has also been increasingly used to promote behavior change [35,36] while sparking debate, especially when considering the abundance of false and misinformation [37]. It is vital to consider the significance of social media to improve vaccine uptake in LMICs.

Promoting health care through various platforms and to youth in LMICs may improve vaccination uptake. Therefore, as part of the Bambisana Project, which includes a diverse and multidisciplinary team comprising social and behavioral scientists, biostatisticians, population health surveillance specialists, health communications researchers, community influence and engagement practitioners, and marketing and communication strategists, we aim to:

1. Evaluate the impact of influencers online, offline, and in collaboration, on influenza vaccine uptake, with a specific focus on young people, and assess the additional value added by social media engagement.
2. Design and implement an integrated communications strategy centered around community influencers and social media to increase the uptake of influenza vaccines, particularly among younger populations who are likely unemployed, partially or informally employed, and living in Soweto (South-Western Townships), South Africa.
3. Explore motivations and barriers to health priorities, and to influenza vaccination, through the use of microinfluencers both online and offline in Soweto, South Africa.

The outcomes of the project will include increased influenza vaccination rates at the local health care clinics linked to campaign interventions, alongside an improved attitude toward the importance of vaccination in the target audience—this will be assessed using the Vaccine Confidence Index scale [38] and measured against the most recent Vaccine Confidence Index data from South Africa [39]. In addition, the project will provide important health systems-strengthening recommendations, which could potentially be implemented within the current infrastructure.

## Methods

### Research Design

The primary research aim uses a prospective mixed methods study design, including qualitative and quantitative data collection, to assess the effects of community and social media interventions to increase influenza vaccine uptake among youth in Soweto, South Africa. During the intervention phase, surveys will be conducted among potential participants attending study clinics who accepted or declined influenza vaccination.

### Study Setting

The study will be conducted in Soweto, which is a congregation of 29 townships within the Johannesburg Metropolis in South Africa [40]. Soweto is one of the poorest areas of Johannesburg with high rates of youth unemployment which is at 46.3% among the 15- to 34-year age group [41].

### Sampling

**Figure 1:**
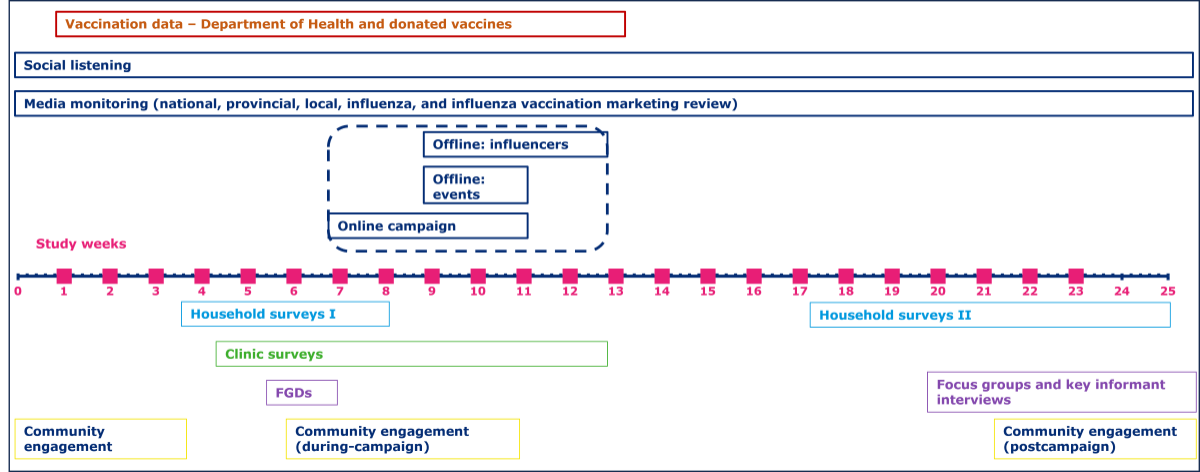
Project timeline: data points and study design. Flu: influenza; FGD: focus group discussion.

The Bambisana Project leverages the Soweto Health and Demographic Surveillance System, led by Wits Vaccines and Infectious Diseases Analytics Research Unit since 2017 and part of the Child Health and Mortality Prevention Surveillance Programme. The Soweto Health and Demographic Surveillance System has been providing access to longitudinal health and demographic data for specific enumerated areas of Soweto and the South of Johannesburg, since 2017 [42]. The study population includes young adults aged 18-34 years from four Health Demographic Surveillance System (HDSS) economically marginalized and geographically distinct Soweto communities namely Phiri, Senaoane, and Mapetla, Mofolo and Meadowlands, Thulani, and neighboring Thembelihle. Adults (18 y and older), living in these clusters or accessing clinic services in the following five study clinics: Senaoane Clinic, Mofolo Community Health Centre, Meadowlands Zone 2 Clinic, Siphumlile Clinic, and Thembelihle Clinic are deemed eligible for the study. Table 1 below shows the population of HDSS communities that will comprise our study clusters while Figure 1 presents the study design.

**Table 1:** Health and Demographic Surveillance System (HDSS) cluster summary data.

Cluster number	Communities	Total population	Youth 18-34 years	Number of households with youth	Total number of households
Cluster 1	Mofolo and Meadowlands	20,986	5824	3478	5912
Cluster 2	Phiri, Senaoane and Mapetla	23,805	6801	4037	6626
Cluster 3	Thulani	23,711	6848	4072	6262
Cluster 4	Thembelihle	19,449	5648	3831	6586

### Community Engagement

The Bambisana (an isiZulu word, which means to work together) study leverages active, long-standing, dynamic community-level relationships and partnerships in accessing community insights at the household and local clinic level. The Wits Vaccines and Infectious Diseases Analytics social behavioral sciences team has longstanding relationships through Community Advisory Boards (CAB) and extensive community engagement in support of health research objectives throughout Soweto. CAB members are individuals residing in the HDSS-enumerated areas, who have been identified as active citizens in their communities. There are standing quarterly CAB meetings with the study team and members; however, these often increase in relation to shifting project activities. Consultations span project development, implementation, and community feedback sessions at the end of data collection, analysis, and interpretation.

### Formative Qualitative Data Collection

The qualitative component includes 16 focus group discussions (FGDs) stratified by age group (18-24 y and 25-34 y), sex (male and female) and community (2 per cluster). These approaches aim to gather contextual data essential for understanding the factors influencing young people’s decisions regarding influenza vaccination, including both motivators and barriers.

Study staff will purposively select and recruit young people for FGDs and key stakeholders from the community for key informant interviews (KIIs). FGD recruitment will be iterative until data saturation is achieved [43]. All study participants will be recruited through the CAB members who work with HDSS communities. Trained and experienced qualitative researchers fluent in local languages spoken in Soweto and Thembelihle (including isiZulu and Sesotho) will conduct the qualitative research using the participants’ language of choice. The FGDs (16 FGDs; 8-12 participants per FGD) and KIIs (20; 10 male and 10 female participants) will be audio-recorded after written informed consent is obtained.

### Intervention

The intervention will be delivered through a public health messaging campaign in targeted clusters or “subplaces” in Soweto, leveraging microinfluencers to provide information and motivate positive attitudes toward vaccination without a mandate in currently unvaccinated populations. The aim of the campaign is to create a behavioral change action: to go to your local clinic to get vaccinated. This has the secondary aim of highlighting the extent to which such intervention might be possible on wider issues of public health in the future. Secondary to this is the desire to investigate the value-add of social media targeting wider forms of engagement, such as that which occurs in communities offline.

The campaign will be timed with points when the clinic is well-supplied and prepared for such a public drive. The project will mitigate the risk of potential vaccine shortages by making vaccine donations to partner clinics. The study will center around the 2023 national influenza vaccination drive. Vaccines are generally provided in a very short and sporadic supply to the local community clinics, so supplying vaccines is also a part of the objective to make a community-level impact as part of the project.

The implementation of the intervention will be guided by information obtained after conducting community engagement discussions, which would be used to design the messaging and communication strategy. Surveys will be administered before, during, and following the communication interventions to establish baseline, midline, and end-line measures for evaluating the intervention’s impact. This will help assess the effectiveness of four distinct approaches aimed at boosting influenza vaccine uptake among youth across clinics in four diverse HDSS clusters.

1. Standard of care: Vaccine made available with standard government promotional activity without support from the project (ie, based on standard government drive).
2. Social media: Social media campaign pushed to the target audience to highlight availability and drive to get vaccinated.
3. Microinfluencers on and offline: The social media campaign will be amplified through a coordinated effort by microinfluencers in their online and offline networks.
4. Offline microinfluencers: Nonsocial media focused; offline microinfluencer-led engagement within communities.

The intervention components are detailed below:

1. Social media campaign to highlight availability and drive to get vaccinated pushed to the target audience (18-34 y) in 4 distinct geographic areas. Using Meta platforms Facebook and Instagram, along with other relevant social media channels identified through community engagement and marketing insights, we will disseminate information through static images, carousel posts, and video posts to promote the availability of, education around, and motivation to get vaccinated. Paid advertising on Meta will target people aged 18-34 years in two of the four distinct geographic study clusters.

2. Microinfluencers on and offline: the social media campaign will be amplified through a coordinated effort by microinfluencers in their online and offline networks. We will use community influencers—people with traditional influence such as community leaders, religious leaders, elders, and key business leaders—to amplify our social media campaign. They will engage their online and offline networks in coordination with our social media campaign to promote vaccine availability and encourage vaccination.

3. Nonsocial media offline microinfluencers: nonsocial media focused; offline microinfluence led engagement within communities. In these communities, our community influencers will focus only on offline engagement and will be specifically instructed to not do anything on social media. Using interpersonal conversations, and small-scale community events to share the importance of vaccines, they will encourage people to get vaccinated.

### Quantitative Data Collection

Quantitative data collection will include five survey data points as outlined below.

All surveys will be developed using the REDCap (Research Electronic Data Capture; Vanderbilt University) software, a computer-assisted survey platform with built-in data encryption [44]. All surveys will be completed using dedicated study tablets, which means participants will not be required to use their internet data. In addition, we will use a data-free survey completion option. The survey links will also be sent to participants who wish to complete the surveys on their own devices. There will be no data costs for participants for completing the assessments. Participants will be reimbursed ZAR 50 (US $3) in airtime toward their time.

Household surveys include 2-time points of data collection, a pre- and posttest design, as described below.

#### Sampling

Households were chosen through a simple random sampling method, and eligible participants were enrolled as follows: a minimum of 800 adult men and women aged 18 years and older, with 400 designated for the pretest and 400 for the posttest. At least 100 households with index participants aged 18-34 years will be approached per community. Nonindex participants include other adults in the households who are aged 18-34, 35-49, and 50 years and older who will also be invited to complete surveys.

#### Pre- and Posttest Surveys

Using a random sample of households having youth participants (18-34 y) generated from the HDSS database, adults will be approached in their households to complete a 15-minute online pretest assessment survey (Multimedia Appendix 1) to measure: sociodemographics (age, gender, food security, and potential loss of income), knowledge of influenza vaccines and benefits, health beliefs, and sources of health information (social media, television, radio, friends, and family), and vaccine confidence [45]. The same households will be invited to participate in the posttest household survey (Multimedia Appendix 2). The posttest household survey will be conducted after the study campaign intervention.

Clinic survey data collection with vaccinees (Multimedia Appendix 3) and those who declined vaccination (Multimedia Appendix 4) are described below.

All adults (older than 18 years) who received an influenza vaccination will be approached; as such, numbers may vary as this depends on who elects to vaccinate. However, a total of at least 400 (n=100 per community) youth (aged 18-34 y) participants who go to the intervention clinics will be purposively approached to complete a postvaccination assessment. The study team will work closely with health care workers at public health clinics in the study communities. Health care workers will refer patients after having received the influenza vaccine, on-site at the clinics. These participants will be invited to complete a brief survey using the same measures as specified for the pretest survey. The data will be covered as part of the project expenses. There will be no data costs for participants to complete the assessments. Participants will be reimbursed ZAR 50 (US $3) in airtime toward their time.

Additional measures will assess motivations for influenza vaccines, where participants heard about this or other drives for the influenza vaccination, if participants had seen any online messaging driving them to get the influenza vaccine or been exposed to specific community engagement activities, and whether it had influenced their decision to vaccinate, and online impressions, for example, video views rates (dependent on final campaign strategy and media mix) and their impact on vaccinations in that region. The uptake of influenza vaccines will be tracked in the 4 clusters across the study period.

Clinic attendees who decline vaccination will also be approached to complete surveys. These participants will be aged 18 years and older. Health care workers will refer patients who were offered vaccination as part of their clinic attendance but who declined vaccination. Those participants who agree to participate will be invited to complete a brief survey to understand the reasons for declining vaccination. The survey measures will include: if and where participants heard about this drive for the influenza vaccination, if participants had seen any online messaging driving them to get the influenza vaccine or been exposed to specific community engagement activities, and whether it had influenced their decision to vaccinate.

As with all the surveys, the data costs will be covered as part of the project expenses. There will be no data costs to participants for completing the assessments. Participants will be reimbursed ZAR 50 (US $3) airtime toward their time.

#### Qualitative Evaluation

Additional FGDs (Multimedia Appendix 5), including 10-12 participants per FGD, will be conducted: 8 midway through the intervention (during vaccination season) and 8 post intervention. These FGDs will be mixed gender and will be stratified by age group (18-24, 25-34 y), and community (2 per cluster). Key informant interviews (KIIs; n=20; Multimedia Appendix 6) with key influencers including, religious leaders, traditional healers, and youth leaders, as well as other influencers that would be identified through community engagement and communications activities.

### Data Analysis

#### Qualitative Data Preparation and Analysis Plan

FGDs and KII recordings will be transcribed verbatim and translated into English for analysis. Transcripts will be analyzed using a framework analysis approach. Framework thematic analysis provides a highly systematic method of categorizing and organizing data according to key themes, concepts, and emergent categories in grids or matrices [46]. Coding will follow a combined approach, ie, both deductive based on our hypothesized themes and inductive (data-driven) to allow for emerging themes. Priori coding based on the interview guide questions will be used to generate an initial coding framework [47]. After developing the initial coding framework, 2 researchers will read through 2 transcripts independently and code them line by line, to identify patterns and themes in the data [47]. This will allow us to further refine the domains and categorize the codes into themes and subthemes. Codes will then be captured using Dedoose, a qualitative data analysis software, and each transcript will be coded in Dedoose by at least 2 researchers and assessed for consistency in the coding of the first 5 transcripts [48]. The research team will convene to review the initial codes and apply general categories that will make up the analytic framework. Any coding disagreements will be discussed until an agreement is reached. This will allow for the finalization of the codebook, which will then be applied to the remaining transcripts. Rigor will be ensured through peer debriefing, triangulation with quantitative data, and member-check presentations with key stakeholders [49].

#### Statistical Analysis Plan

We will use descriptive statistics to describe study variables including knowledge of influenza vaccines, experiences with influenza vaccines, stigma, and vaccine confidence. Reliability assessments using the Cronbach alpha test will be evaluated for scale measures. Scales with poor reliability scores will be calibrated using factor analysis. Data will be analyzed overall and stratified by community. Frequencies will be determined for categorical measures whereas means (SD) and medians (IQR) will be determined for continuous measures. Pairwise comparisons of categorical measures between communities will be evaluated by the chi-square or Fisher exact test as appropriate. Similarly, pairwise continuous measures will be analyzed by the 2-sample *t* test, if satisfying normality distribution conditions or the Wilcoxon Mann-Whitney test, if nonnormally distributed. Normality will be assessed by the Shapiro-Wilks method. Drivers of knowledge, stigma, and experiences of influenza vaccines will be determined by logistic regression for binary outcomes and linear regression for continuous outcomes. Multivariable analysis will be carried out to adjust for potential confounders. Model fit statistics will be assessed on all multivariable regression models to ensure the validity of the findings. Data analyses will be conducted using SAS Enterprise (Guide 7.15; SAS Institute) [50].

### Bias Correction

While the study is designed to focus on four relatively distinct geographic areas, due to the nature of social media and health care–seeking behaviors in the broader community of Soweto, it is possible that members from the intervention communities may interact with those not receiving the intervention. While potential interaction cannot be prevented entirely, research questions for all participants will include questions about exposure to intervention components and other potentially similar national or localized campaigns. In addition, a phased delivery of the interventions to ensure the offline intervention is not biased by the online intervention should help mitigate this.

It is also possible that those exposed to the interventions may seek to be vaccinated in clinics that are not included in the study’s geographically defined target areas. An effort will be made to collaborate with the Department of Health to broadly understand influenza vaccinations during the campaign season, in broader Soweto and areas where residents typically seek health care. Vaccine and vaccination sentiments broadly in Soweto and South Africa will also be tracked during the course of the project for context and monitoring of potential risk.

### Ethical Considerations

All research procedures were approved by the University of the Witwatersrand Human Research Ethics Committee (Medical; M230254) and the local Department of Health. Participants will be reimbursed ZAR 50 (US $3) airtime for survey completion and ZAR 150 (US $8) for the FGDs and the KIIs. This study will be conducted in accordance with the World Medical Association Declaration of Helsinki’s ethical principles for research involving human subjects and will align with the guidelines of the Protection of Personal Information Act (POPIA) 4 of 2013. A copy of the information leaflet detailing the nature and processes involved in the study will be given to the participants. Written informed consent will be obtained from participants before participation, and audio recording of KIIs and FGDs. All information about participants will be confidential and protected, and data dissemination will not include any identifiable information.

## Results

As of May 2024, all data collection is complete, with data analyses and preparation of peer-reviewed publications in progress. The results will encompass qualitative analysis of factors influencing influenza vaccine uptake, a pretest-posttest evaluation of interventions, and campaign intervention methodology along with a longitudinal household investigation of changes in perceptions and attitudes toward influenza vaccination. The first results are expected to be submitted for publication by November 2024.

## Discussion

### Principal Findings

This study will provide critical insights for improving the effectiveness of influenza vaccination campaigns in South Africa. Leveraging micro- and nanoinfluencers on social media, and collaboration with community influencers has been proven to reach and motivate target groups with low vaccine uptake [51]. This study will provide valuable insights into the use of microinfluencers online and offline to reach economically marginalized communities to make positive health decisions where the personal risk perception from not doing so is low in the absence of any mandated reasons to do so. It will also provide an assessment of the differing impact of campaign approaches and the value of the amplification of voices into online social networks for health outcomes.

### Dissemination Plan

Knowledge dissemination will include clear research into action goals through community-based gatherings and activations, radio, and social media–driven activities to key audiences. A strongly connected dissemination platform will be developed by starting with key partners attending an initial study planning meeting, including collaborators, scientists, health policy makers, and representatives from local groups and CAB to recruitment sites. A dedicated website will promote the study and health awareness. A results dissemination group comprising youth CABs and stakeholders will develop knowledge dissemination material for sharing with study participants, stakeholders, and the wider community. Results will be disseminated through press releases, community meetings, social media, and the website. This will be accompanied by scientific presentations to academia, fellow practitioners, and policy makers, publications in open-access peer-reviewed journals, and national and international meetings. Results dissemination meetings will be convened for all study communities and the CAB.

### Conclusions

Enhancing perceptions of, bolstering confidence in, and fostering uptake of vaccination relies heavily on the efficacy of yearly influenza vaccination initiatives, personalized education tailored to specific contexts, active community involvement, consistent vaccine availability, and easily accessible, cost-free distribution channels at the local level.

## Appendix

Multimedia Appendix 1 Household pretest survey.

Multimedia Appendix 2 Household posttest survey.

Multimedia Appendix 3 Clinic (vaccinated) survey.

Multimedia Appendix 4 Clinic (unvaccinated) survey.

Multimedia Appendix 5 Semistructured interview guide for focus group discussions.

Multimedia Appendix 6 Semistructured interview guide for key informant interviews.

## Abbreviations

CAB: Community Advisory Board
FGD: focus group discussion
HDSS: Health and Demographic Surveillance System
KII: key informant interview
LMIC: low- and middle-income country
POPIA: Protection of Personal Information Act
REDCap: Research Electronic Data Capture
Soweto: South-Western Township
WHO: World Health Organization

## Data Availability

The datasets generated/analyzed during this study are available from the corresponding author on reasonable request.
