# Supplementary material for: Assessing Community and Social Media Influence to Increase Influenza Vaccine Uptake among Youth in Soweto, South Africa (The Bambisana Study): Protocol for a Mixed Methods Pretest-Posttest Intervention Study
Source: JMIR Res Protoc. 2025 Jun 17;14:e60481. doi: 10.2196/60481 (PMC12214695; doi:10.2196/60481)
Supplement: Multimedia Appendix 2 [file resprot_v14i1e60481_app2.pdf]

Study Title: Assessing community and social media influence: motivating flu vaccination among youth in Soweto

VCFII Post-test (Household) Survey

Greetings.

Through this study, we want to understand how community influence and social media motivate people in making decisions about their health.

The survey will have 11 sections. You will answer the following sections: 2) Socio-Demographics, 3) Health information, 4) Vaccinations and motivations, 5) Sources of information and influence, 6) Potential barriers and risk perception, 7) Attitudes, 8) Internet access and use, and 9) Knowledge of flu and flu vaccination. It will take about 15-20 minutes to complete the survey.

Thank you for agreeing to participate in this study.

Section 1: Pre-interview (entered by the interviewer)	
**Questions**	**Response options**
Time and date	Automated REDcap response option
Participant ID	
Name of the Cluster	1) Senaoane<br>2) Phiri<br>3) Mapetla<br>4) Meadowlands Zone 4<br>5) Meadowlands Zone 5<br>6) Mofolo<br>7) Thulani<br>8) Thembelihle
Which clinic do you usually go to?	1) Senaoane Clinic<br>2) Meadowlands Zone 2 Clinic<br>3) Mofolo Community Health Centre<br>4) Siphumulile (Thulani) Clinic<br>5) Thembelihle Clinic<br>6) Other (specify)
How long does it take you to get to the clinic?	1) Less than 10 minutes<br>2) 10-20 minutes<br>3) 21-30 minutes<br>4) More than 30 minutes
Which language do you speak the most?	1) IsiZulu<br>2) Sesotho<br>3) IsiXhosa<br>4) Setswana<br>5) Xitsonga<br>6) Tshivenda<br>7) Sepedi<br>8) IsiNdebele<br>9) SiSwati

	10) English<br>11) Afrikaans<br>12) Other (please specify)

Section 2: Socio-demographics (The rest of the survey can be completed by the participant with interviewer available to answer questions if needed)	
What is your age?	Numerical value <18 years
What is your date of birth?	[DDMMYYYY]
What is your sex?	1) Male<br>2) Female<br>3) Other (please specify)
What is your race?	1) Black<br>2) Coloured<br>3) Indian<br>4) White<br>5) Other (please specify)
Where do you live?	1) Senaoane<br>2) Phiri<br>3) Mapetla<br>4) Meadowlands Zone 4<br>5) Meadowlands Zone 5<br>6) Mofolo<br>7) Thulani<br>8) Thembelihle<br>9) Other (please specify)
What is your relationship status?	1) Single<br>2) Married/Living as partners<br>3) Divorced<br>4) Other (please specify)
Do you have children?	1) Yes<br>2) No<br><br>If yes,<br>1) Yes, I have children of primary or high-school age living with me<br>2) Yes, I have children of primary or high-school age, living elsewhere<br>3) Yes, I have adult children
Are you currently attending school?	1) Yes<br>2) No<br><br>If Yes, where are you studying?<br>1) High school<br>2) Further Education and Training (FET) College<br>3) Higher Education Institution<br>4) Other (please specify)

What is the highest level of education that you have completed?	1) School not attended<br>2) Primary school (Grade 7 or below)<br>3) High school (not completed)<br>4) Matric<br>5) Post-matric (diploma, bachelor's degree, post-graduate degree)
What is your current employment status?	1) Permanently employed<br>2) Employed part-time<br>3) Self-employed<br>4) Informally employed, piece jobs<br>5) Unemployed and looking for work<br>6) Unemployed and not looking for work<br>7) Other (please specify)
Do you have a medical aid or hospital plan?	1) Yes<br>2) No<br>3) Other (please specify)
Do you currently receive a social grant?	1) Yes<br>2) No<br>If Yes, which of the following social grants do you receive?<br>1) Child support grant<br>2) Foster child grant<br>3) Old Age Pension<br>4) Disability<br>5) COVID-19 R350 Social Relief Grant<br>6) Other (please specify)
Does someone in your household currently receive a social grant?	1) Yes<br>2) No<br>If Yes, which of the following social grants does your family member receive?<br>1) Child support grant<br>2) Foster child grant<br>3) Old Age Pension<br>4) Disability<br>5) COVID-19 R350 Social Relief Grant<br>6) Other (please specify)

Choose the options that best describe the housing that you currently live in.

Material of the house	1) Concrete or wood<br>2) Mud or thatch
Roof material	1) Tiles or galvanized iron or concrete<br>2) Mud or thatch or plastic
Type of lighting	1) Electricity or gas<br>2) Candle or wood
Source of water	1) Piped into dwelling or borehole with pump or protected dug well<br>2) Pond or unprotected well

Number of people sharing a room in the house	1) 5 or fewer people per room<br>2) 6 or more people per room	
Toilet facilities	1) Flush or ventilated improved latrine<br>2) Open pit or none (bush field)	

Which of the following do you have in your household?

Sewing machine [YES/NO]
Radio [YES/NO]
TV [YES/NO]
Stove [YES/NO]
Fridge [YES/NO]
Mobile phone [YES/NO]
Bicycle [YES/NO]
Motorbike [YES/NO]
Car [YES/NO]
Computer [YES/NO]

Section 3: Health information	
Do have any of these health conditions?	1) Asthma/Chronic Obstructive Pulmonary Disease [YES/NO]<br>2) Hypertension [YES/NO]<br>3) HIV [YES/NO/Not willing to answer]<br>4) Diabetes [YES/NO]<br>5) Chronic Heart Disease [YES/NO]<br>6) Chronic Kidney Disease [YES/NO]<br>7) Cancer [YES/NO]<br>8) Tuberculosis [YES/NO]<br>9) Obesity [YES/NO]<br>10) Other (please specify)<br>11) No, I don't have any existing health conditions
Height in cm	____._/ Don't know
Weight in kilograms	____._/Don't know

Section 4: Vaccinations and motivations	
Please indicate when you remember receiving flu vaccinations	1) 2023 (this year)<br>2) 2022<br>3) 2021<br>4) 2020<br>5) 2019<br>6) 2018<br>7) Before 2018<br>8) I have had flu vaccinations, but can't remember when<br>9) Never had a flu vaccine before
Where have you received your flu vaccine/s in the past?	1) Local clinic<br>2) Public hospital<br>3) Private Doctor

Select all that apply.	4) Private hospital<br>5) Private pharmacy (like Clicks or Dischem)<br>6) Place of work<br>7) Other (please specify)

There is difference between COVID-19 and flu.
Flu (flu) and COVID-19 are both contagious respiratory illnesses, but they are caused by different viruses. COVID-19 is caused by infection with a coronavirus (SARS-CoV-2)first identified in 2019. Flu is caused by infection with a flu virus (flu viruses). COVID-19 can cause more severe illness in some people compared to people with flu. People infected with COVID-19 may take longer to show symptoms and may be contagious for longer periods of time.

The flu vaccine is generally available in private pharmacies to anyone at a cost, and at some locations for free. The Department of Health usually targets healthcare workers, individuals aged 65 years and older, individuals with cardiovascular disease and individuals with immunosuppressive conditions and pregnant women.

Please indicate the extent to which you agree or disagree with the following statements:

I did not know there is a difference between COVID-19 and flu	1) Strongly agree<br>2) Agree<br>3) Neutral<br>4) Disagree<br>5) Strongly disagree
Flu and COVID-19 are the same	1) Strongly agree<br>2) Agree<br>3) Neutral<br>4) Disagree<br>5) Strongly disagree
There is not enough information out there about the difference between flu and COVID-19 vaccines	1) Strongly agree<br>2) Agree<br>3) Neutral<br>4) Disagree<br>5) Strongly disagree

Were you aware that flu vaccines were available for free to anyone in your community this season?<br><br>(Meadowlands, Phiri, Thembelihle, Siphumlile Clinics, and Mofolo Community Health Centre offered free vaccines to anyone, regardless of	1) Yes<br>2) No<br><br>If yes, please tell us how you knew:<br><br>1) Word of mouth (friends, family, community)<br>2) I saw the Bambisana campaign adverts online or on social media<br>3) I saw an outdoor Bambisana campaign activity in Meadowlands or Senaoane

comorbidities or age, during May and June)	4) Someone from the Bambisana campaign approached me in the community to share information or a leaflet<br>5) A community leader or influential person in my life mentioned it<br>6) I don't know<br>7) Other (please specify)
If 'yes' to S4:Q1 (current year vaccinated):	
Did you have any concerns about getting the flu vaccine?	8) Yes<br>9) No<br><br>If yes, please select all that apply:<br><br>1) Effectiveness of the vaccine<br>2) Immediate side effects<br>3) Long-term health effects<br>4) Don't see the need for the vaccine<br>5) I can't afford it (time or cost)<br>10) Other (specify)
Why did you get vaccinated (this year)?<br>Select all that apply	1) To protect my health<br>2) To reduce the severity of flu sickness<br>3) To lower the chances of getting flu<br>4) Because others encouraged me to get vaccinated<br>5) To protect the health of family<br>6) To protect the health of people in my community<br>7) To avoid getting sick so I can continue to go to work or school<br>8) To continue social activities without getting sick<br>9) To be able to travel without getting sick<br>10) Other (specify)
What is the **most** important reason you got vaccinated this year?<br><br>**Select one option**	1) To protect my health<br>2) To reduce the severity of flu sickness<br>3) To lower the chances of getting flu<br>4) Because others encouraged me to get vaccinated (please specify)<br>5) To protect the health of family<br>6) To protect the health of people in my community<br>7) To avoid getting sick so I can continue to go to work or school<br>8) To continue social activities without getting sick<br>9) To be able to travel without getting sick<br>10) Other (specify)

Who, if anyone, convinced you to get vaccinated?<br><br>Select all that apply	1) Family/friends<br>2) Influential community members<br>3) Traditional healers<br>4) NPOs or community support organisations<br>5) Religious leaders<br>6) Local clinic nurse<br>7) Local clinic doctor<br>8) People I follow on social media<br>9) My employer and/or colleagues or school<br>10) Scientists and other experts<br>11) Government and/or politicians (including local community councillors)<br>12) Other (please specify)
What else helped you decide to get vaccinated this year?<br><br>Please select all that apply	1) I saw or heard a flu vaccine advert (online e.g. website or social media, or offline eg. a flyer or poster)<br>2) I saw/was part of a flu vaccine community event<br>3) I heard about a flu vaccine campaign, advert or event from someone I know<br>4) I heard about a flu vaccine through campaign, advert or event in the community (e.g. church, social centre etc)<br><br>(Please tell us what they said about the flu vaccine)<br><br>5) I read a news article that reminded or encouraged me to get vaccinated against flu<br><br>(Please tell us what the article said and where you read it)<br><br>6) I had always planned to get vaccinated<br>7) Other (please specify)
If 'No' to S4:Q1 (2023 current year vaccinated):	
Why did you not get vaccinated this year?<br><br>Select all that apply	1) I did not know about the flu vaccine<br>2) I did not have money to pay for the flu vaccine<br>3) I could not afford the transport<br>4) I could not take time off work<br>5) I do not see a reason to get vaccinated<br>6) I do not trust the vaccination<br>7) I am afraid of the immediate side effects<br>8) I would have gone if I had more information<br>9) I did not think I qualify to get the flu vaccination<br>10) I do not trust healthcare workers

	11) Other (specify)
What is the **most** important reason you did not get vaccinated this year?<br>Select one option	1) I did not know about the flu vaccine<br>2) I did not have money to pay for the flu vaccine<br>3) I could not afford the transport<br>4) I could not take time off work<br>5) I do not see a reason to get vaccinated<br>6) I do not trust the vaccination<br>7) I am afraid of the immediate side effects<br>8) I would have gone if I had more information<br>9) I did not think I qualify to get the flu vaccination<br>10) I do not trust healthcare workers<br>11) Other (specify)
If 'Yes' to any year (S4:Q1) other than current:	
Why did you get vaccinated<br>Select all that apply	1) To protect my health<br>2) To protect my family<br>3) To reduce the severity of flu sickness<br>4) To lower the chances of getting flu<br>5) Because others encouraged me to get vaccinated<br>6) To avoid getting sick so I can continue to go to work or school<br>7) To continue social activities without getting sick<br>8) To be able to travel without getting sick<br>9) To avoid loss of income due to illness<br>10) To have a stronger immune system<br>11) Encouraged or other health institutions<br>12) Encouraged by work<br>13) Other
What is the **most** important reason you got vaccinated in …………?<br><br>**Select one option**	1) To protect my health<br>2) To protect my family<br>3) To reduce the severity of flu sickness<br>4) To lower the chances of getting flu<br>5) Because others encouraged me to get vaccinated<br>6) To avoid getting sick so I can continue to go to work or school<br>7) To continue social activities without getting sick<br>8) To be able to travel without getting sick<br>9) To avoid loss of income due to illness<br>10) To have a stronger immune system<br>11) Encouraged or other health institutions<br>12) Encouraged by work<br>Other
Who previously convinced you to get	1) Family/friends<br>2) Influential community members<br>3) Traditional healers

immunised/vaccinated for flu?<br><br>Select all that apply	4) NPOs or community support organisations<br>5) Religious leaders<br>6) Local clinic nurse<br>7) Local clinic doctor<br>8) Social media influencers<br>9) My employer and/or colleagues or school<br>10) Scientists and other experts<br>11) Government and/or politicians (including local community councillors)<br>12) Other (please specify)

Section 5: Sources of information and influence	
Have you received, seen or heard adverts or information about flu and/or flu vaccines recently (this year)?	1) Yes<br>2) No<br><br>If Yes, please indicate where:<br>1. Text message or SMS<br>2. In newspapers<br>3. On billboards or outdoors<br>4. On TV<br>5. On the radio<br>6. Online (websites, adverts)<br>7. On social media (this question refers to adverts, not personal posts)<br>8. In email newsletters from companies or organisations<br>9. In person in my community eg. community events<br>10. Other (please specify)
What advertising about flu vaccines have you received, seen or heard?<br><br>Select all that apply	1) Advertising by pharmacies (such as Dischem or Clicks)<br>2) Advertising by medical aids<br>3) Department of Health advertising or announcements<br>4) School or workplace messages<br>5) The Bambisana campaign<br>6) Other (please specify)
Did you see this logo or campaign anywhere? Select all that apply<br>[show Bambisana logo]	1) Yes<br>2) No<br><br>If yes, where?<br>3) On social media<br>4) Online (website)

	5) Outdoors at community events in Meadowlands or Senaoane<br>6) I found or someone gave me a flyer<br>7) Other (please specify)
Did the information youreceived through the Bambisana campaign change your views on flu and flu vaccination?	1) Yes<br>2) No<br>3) Neutral<br>4) I don't know
I have recently heard friends, family or colleagues talking about flu and/or flu vaccines	1) Strongly agree<br>2) Agree<br>3) Neutral<br>4) Disagree<br>5) Strongly disagree
The flu vaccine information or adverts I saw this year increased my understanding of flu and the importance of flu vaccination	1) Strongly agree<br>2) Agree<br>3) Neutral<br>4) Disagree<br>5) Strongly disagree
The flu vaccine information or adverts I saw this year made me feel that vaccination is important and necessary	1) Strongly agree<br>2) Agree<br>3) Neutral<br>4) Disagree<br>5) Strongly disagree

Choose the top three: Which platforms do you think provide reliable, trustworthy information…

… about general health?	… about immunisations/vaccinations?
1) Television<br>2) Commercial radio (e.g. Metro FM, YFM, Kaya FM)<br>3) Community radio (e.g. Jozi FM, Alex FM)<br>4) National newspapers (e.g. Sowetan, Sunday Times, City Press, The Star, Daily Sun)<br>5) Local newspapers (Caxton community newspaper, Soweto Urban)<br>6) Government communication (e.g. website, SMS)<br>7) Social media (e.g. Facebook, TikTok, Instagram)<br>8) The Internet (e.g. Google)<br>9) WhatsApp<br>10) Other (please specify)	1) Television<br>2) Commercial radio (e.g. Metro FM, YFM, Kaya FM)<br>3) Community radio (e.g. Jozi FM, Alex FM)<br>4) National newspapers (e.g. Sowetan, Sunday Times, City Press, The Star, Daily Sun)<br>5) Local newspapers (Caxton community newspaper, Soweto Urban)<br>6) Government communication (e.g. website, SMS)<br>7) Social media (e.g. Facebook, TikTok, Instagram)<br>8) The Internet (e.g. Google)<br>9) WhatsApp<br>10) Other (please specify)<br>11) None of these platforms provide reliable information

11) None of these platforms provide reliable information	

Select the top three: Which of the following social media and messaging platforms do you think provide reliable, trustworthy information?

… about general health?	… about immunisation/vaccines?
1) Facebook<br>2) Instagram<br>3) Twitter<br>4) TikTok<br>5) YouTube<br>6) WhatsApp<br>7) Social media or messaging platforms do not provide reliable information about health<br>8) Other (please specify)	1) Facebook<br>2) Instagram<br>3) Twitter<br>4) TikTok<br>5) YouTube<br>6) WhatsApp<br>7) Social media or messaging platforms do not provide reliable information about vaccines or immunisations<br>8) Other (please specify)

Select the top three people or organizations that you would go to for reliable, trustworthy information …

… about general health?	… about immunisations?
1) Family/friends<br>2) Influential community members<br>3) Traditional healers<br>4) NPOs or community support organisations<br>5) Religious leaders<br>6) Local clinic nurse<br>7) Local clinic doctor<br>8) Social media influencers<br>9) My employer and/or colleagues<br>10) Scientists and other experts<br>11) Government and/or politicians<br>12) Other (please specify)	1) Family/friends<br>2) Influential community members<br>3) Traditional healers<br>4) NPOs or community support organisations<br>5) Religious leaders<br>6) Local clinic nurse<br>7) Local clinic doctor<br>8) Social media influencers<br>9) My employer and/or colleagues<br>10) Scientists and other experts<br>11) Government and/or politicians<br>12) Other (please specify)

Section 6: Potential barriers and risk perceptions

How easy or difficult is it to find a clinic to get vaccinated for the flu?	1) Very easy<br>2) Easy<br>3) Difficult<br>4) Very difficult
How easy or difficult is it to find transport to the clinic?	1) Very easy<br>2) Easy<br>3) Difficult<br>4) Very difficult
How easy or difficult is it to afford transport to the clinic?	1) Very easy<br>2) Easy<br>3) Difficult<br>4) Very difficult
How easy or difficult is it to find a clinic for vaccination with convenient opening hours?	1) Very easy<br>2) Easy<br>3) Difficult<br>4) Very difficult

Would you have to take time off from work/school to get vaccinated?	1) Yes<br>2) No<br>3) Not applicable<br>If Yes, how easy or difficult is getting time off from work/school to get vaccinated?<br>1) Very easy<br>2) Easy<br>3) Difficult<br>4) Very difficult
Would having to take time off from work/school prevent you from getting vaccinated?	1) Yes<br>2) No<br>3) Not applicable
Would you have to arrange childcare to get vaccinated?	1) Yes<br>2) No<br>3) Not applicable<br>If Yes, how easy or difficult is it to arrange childcare to get vaccinated?<br>1) Very easy<br>2) Easy<br>3) Difficult<br>4) Very difficult
Having to take time off from work or school would prevent me from getting vaccinated (for flu)	1) Yes<br>2) No<br>3) Not applicable

I don't worry about getting sick from colds or the flu	1) Strongly agree<br>2) Agree<br>3) Neutral<br>4) Disagree<br>5) Strongly disagree
Flu is seasonal; I will get better on my own	1) Strongly agree<br>2) Agree<br>3) Neutral<br>4) Disagree<br>5) Strongly disagree
I know the difference between flu and COVID-19 vaccines	1) Strongly agree<br>2) Agree<br>3) Neutral<br>4) Disagree<br>5) Strongly disagree
I prefer the word 'immunisation' when talking about an injection that could prevent an illness or prevent severe illness.	1) Strongly agree<br>2) Agree<br>3) Neutral<br>4) Disagree<br>5) Strongly disagree

Section 7: Attitudes

Indicate to what extent you agree or disagree with the following statements.

"Immunisations are important for children to have"	1) Strongly agree<br>2) Agree<br>3) Neutral<br>4) Disagree<br>5) Strongly disagree
"Immunisations are safe"	1) Strongly agree<br>2) Agree<br>3) Neutral<br>4) Disagree<br>5) Strongly disagree
"Immunisations are effective"	1) Strongly agree<br>2) Agree<br>3) Neutral<br>4) Disagree<br>5) Strongly disagree
"Immunisations are compatible with my religious, personal and philosophical beliefs"	1) Strongly agree<br>2) Agree<br>3) Neutral<br>4) Disagree<br>5) Strongly disagree

Section 8: Internet access and use

Do you have access to the internet?	1) Yes<br>2) No

	If No, what is the reason? (select all that apply)<br>1) I don't own a device that can access the internet<br>2) My cell phone is not a smartphone<br>3) I can't afford data bundles or Wi-Fi<br>4) Other (please specify)<br>If Yes, which device or devices do you use to access the internet? (select all that apply)<br>1) Smartphone<br>2) Tablet<br>3) Laptop/Computer<br>4) Other (please specify)
Where do you access the internet?	1) At home<br>2) At work, for work use only<br>3) At work, for work and/or personal use<br>4) From an internet cafe or public location such as Postnet<br>5) While travelling in a taxi/bus Other (please specify)
How do you connect to the internet?	1) Cell phone contract data package<br>2) General data bundles<br>3) WhatsApp-only data bundles<br>4) Facebook-only data bundles<br>5) Wi-Fi at home<br>6) Public Wi-Fi<br>7) Other (please specify)
Who usually pays for your data?	1) Myself<br>2) Parent<br>3) Boyfriend/girlfriend/partner<br>4) Sibling<br>Other (please specify)
Which social media platforms have you accessed in the past 30 days?	1) Facebook<br>2) Instagram<br>3) TikTok<br>4) Twitter<br>5) YouTube<br>6) Other (please specify)
Which social media platform do you use the most?	1) Facebook<br>2) Instagram<br>3) TikTok<br>4) Twitter<br>5) YouTube<br>6) Other (please specify)
Do you use WhatsApp?	1) Yes<br>2) No<br>If Yes, how do you use WhatsApp?<br>1) Communicating with individuals family, friends or colleagues

	2) Participating in small WhatsApp groups (under 100 members)<br>3) Participating in large WhatsApp groups (over 100 members)<br>4) Broadcasting messages to multiple contacts (eg. for business)<br>If No, how do you communicate with people or groups?<br>1) SMS<br>2) Telephone<br>3) Other platforms (please specify)
Are you a member of any WhatsApp groups?	1) Yes<br>2) No<br>If Yes, what do you use WhatsApp groups for? (select all that apply)<br>1) Messaging friends and family<br>2) Sharing news and content<br>3) Finding employment opportunities<br>4) Neighbourhood watch<br>5) Assessing and sharing health information<br>6) Other (please specify)
On average, how many hours do you spend online? (This excludes WhatsApp)	1) 1-2 hours<br>2) 2-4 hours<br>3) 4-6 hours<br>4) 6-8 hours<br>5) More than 8 hours
During which time of the day?	1) 6 am-12pm<br>2) 12pm-6pm<br>3) 6pm-10pm<br>4) After 10pm

Section 9: Knowledge of flu and flu vaccination	
How often should people get vaccinated against flu?	1) Every year<br>2) Once<br>3) Never<br>4) Don't know<br>5) Other (please specify)
Who should get vaccinated against flu?	1) Everyone<br>2) Adults 65 and older<br>3) Pregnant women<br>4) People with chronic disease<br>5) Healthcare professionals
During which time of the do people in South Africa get sick from flu?	1) All year<br>2) During winter months<br>3) During the summer months<br>4) Other (please)

Indicate to what extent you agree or disagree with the following statements

"Being vaccinated reduces the severity and duration of flu"	1) Strongly agree<br>2) Agree<br>3) Neutral<br>4) Disagree<br>5) Strongly disagree
"Being vaccinated against flu improves immunity"	1) Strongly agree<br>2) Agree<br>3) Neutral<br>4) Disagree<br>5) Strongly disagree
"Severe illness and complication from flu can lead to absence from school or work and affect quality of work."	1) Strongly agree<br>2) Agree<br>3) Neutral<br>4) Disagree<br>5) Strongly disagree
"Severe illness from flu can lead to hospitalisation or even death."	1) Strongly agree<br>2) Agree<br>3) Neutral<br>4) Disagree<br>5) Strongly disagree
"Flu vaccination is effective if someone is already infected with flu"	1) Strongly agree<br>2) Agree<br>3) Neutral<br>4) Disagree<br>5) Strongly disagree

You have reached the end of the survey. Thank you for participating.
