# Supplementary material for: Assessing Community and Social Media Influence to Increase Influenza Vaccine Uptake among Youth in Soweto, South Africa (The Bambisana Study): Protocol for a Mixed Methods Pretest-Posttest Intervention Study
Source: JMIR Res Protoc. 2025 Jun 17;14:e60481. doi: 10.2196/60481 (PMC12214695; doi:10.2196/60481)
Supplement: Multimedia Appendix 3 [file resprot_v14i1e60481_app3.pdf]

Post-test (Clinic vaccinated) Survey

Record ID ______________________

INFORMATION LEAFLET AND INFORMED CONSENT FORM FOR SURVEY:

Assessing community and social media influence: motivating influenza vaccination among youth in Soweto

CO-PRINCIPAL INVESTIGATORS: Dr Nellie Myburgh (Wits VIDA), Prof Janan Dietrich (PHRU)

SUB-INVESTIGATORS: Dr Kimberley Gutu, Catherine Hill, Prof Madhi

SITE ADDRESS: Wits VIDA Research Unit, Nurses Residence, Nurses Home Road, Chris Hani Baragwanath Academic Hospital, Chris Hani Road, Soweto, 1862, Gauteng, South Africa

SITE TELEPHONE NUMBER: +27 11 983 4283

Please read this form carefully and ask the study staff to explain any words or procedures you do not understand.

Good day, my name is.................................... (Insert name of study staff member). I am a study staff member at Wits VIDA, a research Unit at the University of the Witwatersrand. You are invited to participate in a research study on the impact of community influence and social media in motivating flu vaccination efforts in Soweto South Africa. Participants enrolled in this study will be aged 18 years and older and living in Soweto and Thembelihle.

Today we invite you to take part in a short survey which you can complete on your own or a study staff member can help you. I am giving you information about the study and what you will be asked to do if you decide to participate in the study. If you agree to participate in the study, you will be asked to provide written informed consent today.

We will request that we send you a data free survey link that you can complete to confirm your agreement to participate. All participant data will be entered electronically using the link sent to your phone number or on the study tablet.

Your data will be saved and stored in a secure and encrypted online database. We will not use any identifying data (such as your name) in any dissemination of the study findings. We hope that you will agree to share your contact information with our staff member so that they can share the questionnaire link with you.

Your participation in this study is voluntary and you can decide to stop survey completion at any stage. . This study will not lead to any changes in your routine care. You will not be receiving any intervention (vaccine, drug, other) as part of the study. Therefore, there will be no direct benefits or foreseeable risks to you from your participation in this research study. You may experience discrimination from others around you as a result of your participation in the study.

The final data used for the research project will not be linked with your name, contact details or any other personal information about you that could be used to trace your identity. So, your individual identity will be protected.

Wits VIDA will assign a responsible person to use and store the research data in a safe place. The key-coded data obtained from this study will be stored in a secured database located in South Africa. Your personal data will always be handled in accordance with all applicable data protection and privacy laws. All information about you as an individual will be confidential and will be protected. The information will only be communicated to authorized persons who will respect the same confidentiality. Information may also be inspected by the National Health Research Ethics Council (NHREC) and the University of the Witwatersrand, Human Research Ethics Committee (HREC). Any information collected from other physicians will be handled in the same confidential manner as that collected by the study doctor. Data that may be reported in scientific journals will not include any information that identifies you as a participant in this study. Although we will ask participants to maintain confidentiality there is no way to ensure this will be respected.

Data will be archived for 10 years, as per national regulations, and will then be destroyed. Should you decide to withdraw from the study, data collected up until the time of withdrawal will be used in the analyses, but no further data will be collected.

Your participation in this study is entirely voluntary and you can decline to participate or stop at any time, without stating any reason. Your withdrawal will not affect your access to other medical care. You have the right not to participate without giving a reason or penalty or loss of benefits. If you have questions about this study or any problems related to this study, contact the Sub-investigator Dr Nellie Myburgh at the following email:

nellie.myburgh@wits-vida.org or Tel: +2711 983 4283

The study has been structured in accordance with the Declaration of Helsinki (last updated: October 2013), which deals with the recommendations guiding doctors in biomedical research involving human participants. A copy may

be obtained from me should you wish to review it. This clinical study protocol has been submitted to the University of the Witwatersrand, Human Research Ethics Committee (HREC): Medical and written approval has been granted by that committee. If you have any concern/s, please contact the Chairperson of this Committee who is Professor Clement Penny, who may be contacted on telephone number 011 717 2301, or by e-mail on Clement.Penny@wits.ac.za.

By signing below, I hereby confirm that I have been informed about the nature, conduct, benefits, and risks of the study and that:

I have received, read, and understood the above written information I understand the risks and benefits associated with participating in this study I have been given a range of contact details, listed below. If I require further information or become concerned about any aspect of this study, I am free to speak to any of these contacts. Was the patient able to provide written/verbal consent to ALL conditions above? (Only answer 'Yes' if participant agrees to all the conditions of consent. End survey if participant does not provide consent.)

Yes/ No

◯ Yes
◯ No

Participant ______
Signature ______
Name and Surname ______

Study Staff ______
Signature ______
Name and Surname ______

Witness ______
Signature ______
Name and Surname ______

Greetings,

Through this study, we want to understand how community influence and social media motivate people in making decisions about their health.

The survey will have 10 sections. You will answer the following sections: 2) Socio- Demographics, 3) Health information, 4) Vaccinations, 5) Exposure to flu vaccination communications, 6) Potential barriers and risk perception, 7) Attitudes, 8) Internet access and use, 9) Sources of information and influence, and 10) Knowledge of flu vaccination. It will take about 10-15 minutes to complete the survey.

Thank you for agreeing to participate in this study

______

Pre-interview

Time and Date

____

Enter Participant ID

____

Do you have a mobile phone number or alternative number to contact you?

◯ Yes
◯ No

What is your cellphone number?

____

Which cellphone network provider do you use?

◯ Cell C
◯ Vodacom
◯ MTN
◯ Telkom

Name of clinic

◯ Senaoane Clinic
◯ Meadowlands Zone 2 Clinic
◯ Mofolo Community Health Centre
◯ Siphumulile (Thulani) Clinic
◯ Thembelihle Clinic

How long did it take you to get to the clinic today?

◯ Less than 10 minutes
◯ 10-20 minutes
◯ 21-30 minutes
◯ More than 30 minutes

What was the main reason for visiting the clinic today?

◯ For the healthcare needs of my child
◯ For personal healthcare reasons, other than vaccinations
◯ Other (please specify)

Other reason for visiting the clinic today?

____

Which language do you speak the most?

◯ IsiZulu
◯ Sesotho
◯ IsiXhosa
◯ Setswana
◯ Xitsonga
◯ Tshivenda
◯ Sepedi
◯ IsiNdebele
◯ SiSwati
◯ English
◯ Afrikaans
◯ Other (please specify)

Other language do you speak the most ?

____

Socio-demographics
(The rest of the survey can be completed by the participant with interviewer available to assist if needed)

What is your age?

____

What is your gender?

◯ Male
◯ Female
◯ Other (please specify)

Other Gender?

____

What is your race?

◯ Black
◯ Coloured
◯ not Indian
◯ Indian
◯ White
◯ Other (please specify)

Other your race?

____

Where do you live?

◯ Senaoane
◯ Phiri
◯ Mapetla
◯ Meadowlands Zone 4
◯ Meadowlands Zone 5
◯ Mofolo
◯ Thulani
◯ Thembelihle
◯ Other (please specify)

Other Household location

____

What is your relationship status?

◯ Single
◯ Married/Living as partners
◯ Divorced

Do you have children?

◯ Yes, I have children of primary or high-school age living with me
◯ Yes, I have children of primary or high- school age, living elsewhere
◯ Yes, I have adult children
◯ No, I do not have children

Are you currently attending school?

◯ Yes
◯ No

Where are you studying?

◯ High school
◯ Further Education and Training (FET) College
◯ Higher Education Institution
◯ Other (please specify)

Other school currently studying?

____

What is the level of education that you have completed?

◯ School not attended
◯ Primary school (Grade 7 or below)
◯ High school (not completed)
◯ Matric
◯ post-matric (diploma, bachelor's degree, post-graduate degree

What is your current employment status?

◯ Permanently employed
◯ Employed part-time
◯ Self-employed
◯ Informally employed, piece jobs
◯ Unemployed and looking for work
◯ Unemployed and not looking for work
◯ Other (please specify)

Other employment status

____

Do you have a medical aid or hospital plan?

◯ Yes
◯ No
◯ Other (please specify)

Other medical aid or hospital plan?

____

Do you currently receive a social grant?

◯ Yes
◯ No

Which of the following social grants do you receive?

☐ Child support grant
☐ Foster child grant
☐ Old Age Pension
☐ Disability
☐ COVID-19 R350 Social Relief Grant
☐ Other (please specify)

Other source of social grant?

____

Do someone in your household currently receive a social grant?

◯ Yes
◯ No

which of the following social grants do you and/or a family member receive? (Select all that apply)

☐ Child support grant
☐ Foster child grant
☐ Old Age Pension
☐ Disability
☐ COVID-19 R350 Social Relief Grant
☐ Other (please specify)

Other social grants do you and/or a family member receive? __________________________________

Choose the options that best describe the housing that you currently live in.

Material of the house

◯ Concrete or wood
◯ Mud or thatch

Roof material

◯ Tiles or galvanized iron or concrete
◯ Mud or thatch or plastic

Type of lighting

◯ Electricity or gas
◯ Candle or wood

Source of water

◯ Piped into dwelling or borehole with pump or protected dug well
◯ Pond or unprotected well

Number of people sharing a room in the house?

◯ 5 or fewer people per room
◯ 6 or more people per room

Toilet facilities

◯ Flush or ventilated improved latrine
◯ Open pit or none (bush field)

Which of the following do you have in your household?

☐ Sewing machine
☐ Radio
☐ TV
☐ Stove
☐ Fridge
☐ Mobile phone
☐ Bicycle
☐ Motorbike
☐ Car
☐ Computer

Health information

Do you have any of these health conditions?

☐ Asthma/Chronic Obstructive Pulmonary Disease
☐ Hypertension
☐ HIV
☐ Diabetes
☐ Chronic Heart Disease
☐ Chronic Kidney Disease
☐ Cancer
☐ Tuberculosis
☐ Obesity
☐ No, I don't have any existing health conditions
☐ Other (please specify)

Other health conditions

____

Vaccinations

Have you been vaccinated today?

◯ Yes
◯ No

Did a doctor or nurse here at the clinic tell you about the flu vaccine today?

◯ Yes
◯ No, I came to the clinic to get vaccinated for the flu today
◯ Other (please specify)

Other Individual here at the clinic who tells you about the flu vaccine today?

____

From the time you arrived, how long did you have to wait before you were vaccinated?

◯ Less than 10 minutes
◯ 10-20 minutes
◯ 21-30 minutes
◯ More than 30 minutes

Did you have any worries or concerns about getting vaccinated today?

◯ Yes, major concerns Please specify
◯ Yes, minor concerns Please specify
◯ No concerns
◯ I don't know

Please specify major concerns

____

Please specify minor concerns

____

What is the Main reason you chose not to get vaccinated?

◯ I did not think it is necessary
◯ I'm afraid of the immediate side effects
◯ I don't trust vaccines
◯ I don't worry about getting sick from the flu
◯ Other (please specify)

Other reason you chose not to get vaccinated?

____

Please indicate when you remember receiving flu vaccinations ?

◯ 2023 (this year)
◯ 2022
◯ 2021
◯ 2020
◯ 2019
◯ 2018
◯ Before 2018
◯ I have had flu vaccinations, but can't remember when
◯ I have never received a flu vaccine

In the past, where have you received your flu vaccines?

◯ Local clinic
◯ Public hospital
◯ Private Doctor
◯ Private hospital
◯ Pharmacy
◯ Place of work
◯ Other (please specify)

Other place In the past, where have you received your flu vaccines?

____

Exposure to flu vaccination communications and motivations for flu vaccination

Have you seen adverts about flu and/or flu vaccines recently (this year)?

◯ Yes
◯ No

please indicate where ?

☐ In newspapers
☐ On billboards or outdoors
☐ On TV
☐ On the radio
☐ Online (websites, adverts)
☐ On social media channels (this question refers to adverts, not personal posts)
☐ In email newsletters from companies or organisations
☐ Other (please specify)

Other platform.

____

What advertising about flu vaccines have you seen?

☐ Advertising by pharmacies (such as Dischem or Clicks)
☐ Advertising by medical aids
☐ Department of Health
☐ advertising or announcements
☐ School or workplace messages
☐ Other (please specify)

Other advertising about flu vaccines have you seen?

____

Have you heard adverts about flu and/or flu vaccines recently (this year)?

◯ Yes
◯ No

please indicate where?

☐ In newspapers
☐ On billboards or outdoors
☐ On TV
☐ On the radio
☐ Online (websites, adverts)
☐ On social media channels (this question refers to adverts, not personal posts)
☐ In email newsletters from companies or organisations
☐ Other (please specify)

Other platform.

____

What advertising about flu vaccines have you heard?

☐ Advertising by pharmacies (such as Dischem or Clicks)
☐ Advertising by medical aids
☐ Department of Health
☐ advertising or announcements
☐ School or workplace messages
☐ Other (please specify)

Other advertising about flu vaccines have you Heard?

____

What are your reasons for coming to get vaccinated today?

☐ I saw or heard a flu vaccine advert.
☐ I saw/was part of a flu vaccine community event.
☐ I heard about a flu vaccine campaign, advert or event from someone I know and trust.
☐ A family member/friend advised me to do so.
☐ A doctor/nurse advised me to do so.
☐ My religious leader advised me to do so.
☐ A community leader/local organisation advised me to do so.
☐ People in my community advised me to do so.
☐ My employer advised me to do so.
☐ My school/college/university advised me to do so.
☐ I had always planned to get vaccinated.
☐ Other (please specify)

Other reasons for coming to get vaccinated today?

____

Potential barriers and risk perception

	Very easy	Easy	Difficult	Very difficult
How easy or difficult is it to find a site to get vaccinated for the flu?	◯	◯	◯	◯
How easy or difficult is it to find transport to the site?	◯	◯	◯	◯
How easy or difficult is it to afford transport to the site?	◯	◯	◯	◯
How easy or difficult is it to find a vaccination site with convenient opening hours?	◯	◯	◯	◯

Did you have to take time off from work/school to come to the clinic?

◯ Yes
◯ No
◯ Not applicable

	1Very easy	Easy	Difficult	Very difficul
how easy or difficult is it to ge time off from work/school to come to the clinic?	◯	◯	◯	◯

Did you have to arrange childcare to come to the clinic?

◯ Yes
◯ No
◯ Not applicable

	Very easy	Easy	Difficult	Very difficult
how easy or difficult is it to arrange childcare to come to the clinic?	◯	◯	◯	◯

	Strongly agree	Agree	Neutral	Disagree	Strongly disagree
I don't worry about getting sick from cold or the flu	◯	◯	◯	◯	◯
Flu is seasonal; I will get better on my own	◯	◯	◯	◯	◯

Attitudes

	Strongly agree	Agree	Neutral	Disagree	Strongly disagree
"Immunisations are important for children to have	◯	◯	◯	◯	◯
"Immunisations are safe"	◯	◯	◯	◯	◯
"Immunisations are effective"	◯	◯	◯	◯	◯
"Immunisations are compatible with my religious, personal and philosophical beliefs	◯	◯	◯	◯	◯

Internet access and use

Do you have access to the internet?

◯ Yes
◯ No

what is the reason?

☐ I don't own a device that can access the internet
☐ My cell phone is not a smartphone
☐ I can't afford data bundles or Wi-Fi
☐ Other (please specify)

Other reason not having access to internet?

____

which device or devices do you use to access the internet

☐ Smartphone
☐ Tablet
☐ Laptop
☐ Other (please specify)

Other device or devices do you use to access the internet ______________________

Where do you access the internet?

☐ At home
☐ At work, for work use only
☐ At work, for work and/or personal use
☐ From an internet cafe or public location such as Postnet
☐ While travelling in a taxi/bus
☐ Other (please specify)

Other access into the internet?

____

How do you connect to the internet?

○ Cell phone contract data package
○ General data bundles
○ WhatsApp-only data bundles
○ Facebook-only data bundles
○ Wi-Fi at home
○ Public Wi-Fi
○ Other (please specify

Other connectivity to the internet?

____

Who usually pays for your data?

○ Myself
○ Parent
○ Boyfriend/girlfriend/partner
○ Sibling
○ Other (please specify)

Other payments of your data? ______________________

Which social media platforms have you accessed in the past 30 days?

☐ Facebook
☐ Instagram
☐ TikTok
☐ Twitter
☐ YouTube
☐ Other (please specify)

Other social media platforms have you accessed in the past 30 days?

____

Which social media platform do you use the most?

☐ Facebook
☐ Instagram
☐ TikTok
☐ Twitter
☐ YouTube
☐ Other (please specify)

Other social media platform do you use the most?

____

Do you use WhatsApp?

◯ Yes
◯ No

How do you use WhatsApp?

◯ Communicating with individuals family, friends or colleagues
◯ Participating in small WhatsApp groups (under 100 members)
◯ Participating in large WhatsApp groups (over 100 members)
◯ Broadcasting messages to multiple contacts (eg. for business)

how do you communicate with people or groups?

◯ SMS
◯ Telephone
◯ Other platforms (please specify)

Other means do you communicate with people or groups?

____

Are you a member of any WhatsApp groups?

◯ Yes
◯ No

what do you use WhatsApp groups for? (select all that apply)

☐ Messaging friends and family
☐ Sharing news and content
☐ Finding employment opportunities
☐ Neighbourhood watch
☐ Assessing and sharing health information
☐ Other (please specify)

Other use of WhatsApp groups

____

On average, how many hours do you spend online? (This excludes WhatsApp)

◯ 1-2 hours
◯ 2-4 hours
◯ 4-6 hours
◯ 6-8 hours
◯ More than 8 hours

During which time of the day?

◯ 6 am-12pm
◯ 12pm-6pm
◯ 6pm-10pm
◯ After 10pm

Sources of information and influence

Choose the top three: Which platforms do you think provide reliable, trustworthy information...
About general_health About immunisations

______

______

______

______

Select the top three that apply: Which of the following social media and messaging platforms do you think provide reliable, trustworthy information ...
About general health About vaccine

______

______

______

______

Select the top three people that you would go to for reliable, trustworthy information
About general health? Other individual about general health?

______

______

______

______

Knowledge of flu vaccination

How often should people get vaccinated against flu?

☐ Every year
☐ Once
☐ Never
☐ Don't know
☐ Other (please specify)

Other period should people get vaccinated against flu?

____

Who should get vaccinated against flu?

☐ Everyone
☐ Adults 65 and older
☐ Pregnant women
☐ People with chronic disease
☐ Healthcare professionals

During which time of the year do people in South Africa get sick from flu?

◯ All year
◯ During winter months
◯ During the summer months
◯ Other (please specify)

Other time of the year do people in South Africa get sick from flu?

____

Indicate to what extent you agree or disagree with the following statements

	Strongly agree	Agree	Neutral	Disagree	Strongly disagree
"Being vaccinated reduces the severity and duration of flu"	◯	◯	◯	◯	◯
"Being vaccinated against flu improves immunity"	◯	◯	◯	◯	◯
"Severe illness and complication from flu can lead to absence from school or work and affect quality of work."	◯	◯	◯	◯	◯
"Severe illness from flu can lead to hospitalisation or even death."	◯	◯	◯	◯	◯
"Flu vaccination is effective if someone is already infected with flu"	◯	◯	◯	◯	◯

You have reached the end of the survey.

Completed By:

____
