# Supplementary material for: Assessing Community and Social Media Influence to Increase Influenza Vaccine Uptake among Youth in Soweto, South Africa (The Bambisana Study): Protocol for a Mixed Methods Pretest-Posttest Intervention Study
Source: JMIR Res Protoc. 2025 Jun 17;14:e60481. doi: 10.2196/60481 (PMC12214695; doi:10.2196/60481)
Supplement: Multimedia Appendix 4 [file resprot_v14i1e60481_app4.pdf]

Study Title: Assessing community and social media influence: motivating influenza vaccination among youth in Soweto
VCFII Post-test (Clinic Unvaccinated) Survey

Greetings,

Through this study, we want to understand how community influence and social media motivate people in making decisions about their health.

The survey will have 10 sections. You will answer the following sections: 2) Socio-Demographics, 3) Health information, 4) Vaccinations, 5) Exposure to flu vaccination communications, 6) Potential barriers and risk perception, 7) Attitudes, 8) Internet access and use, 9) Sources of information and influence, and 10) Knowledge of flu vaccination. It will take about 10-15 minutes to complete the survey.

Thank you for agreeing to participate in this study.

Section 1: Pre-interview (entered by the interviewer)	
Questions	Response options
Time and date	Automated REDcap response option
Enter Participant ID	Free test response
Name of the clinic	1) Senaoane Clinic<br>2) Meadowlands Zone 2 Clinic<br>3) Mofolo Community Health Centre<br>4) Siphumulile (Thulani) Clinic<br>5) Thembelihle Clinic
How long did it take you to get to the clinic today?	1) Less than 10 minutes<br>2) 10-20 minutes<br>3) 21-30 minutes<br>4) More than 30 minutes
What was the main reason for visiting the clinic today?	1) For the healthcare needs of my child<br>2) For personal healthcare reasons, other than vaccinations<br>3) Other (please specify)
Which language do you speak the most?	1) IsiZulu<br>2) Sesotho<br>3) IsiXhosa<br>4) Setswana<br>5) Xitsonga<br>6) Tshivenda<br>7) Sepedi<br>8) IsiNdebele<br>9) SiSwati<br>10) English

	11) Afrikaans<br>12) Other (please specify)

Section 2: Socio-demographics (The rest of the survey is completed by the participant with interviewer available to assist if needed)	
What is your age?	Free text response
What is your sex?	1) Male<br>2) Female<br>3) Other (please specify)
What is your race?	1) Black<br>2) Coloured<br>3) Asian, not Indian<br>4) Indian<br>5) White<br>6) Other (please specify)
Where do you live?	1) Senaoane<br>2) Phiri<br>3) Mapetla<br>4) Meadowlands Zone 4<br>5) Meadowlands Zone 5<br>6) Mofolo<br>7) Thulani<br>8) Thembelihle<br>9) Other (please specify)
What is your relationship status?	1) Single<br>2) Married/Living as partners<br>3) Divorced
Do you have children?	1) Yes, I have children of primary or high-school age living with me<br>2) Yes, I have children of primary or high-school age, living elsewhere<br>3) Yes, I have adult children<br>4) No, I do not have children
Are you currently attending school?	1) Yes<br>2) No<br>If Yes, where are you studying?<br>1) High school<br>2) Further Education and Training (FET) College<br>3) Higher Education Institution<br>4) Other (please specify)
What is the level of education that you have completed?	1) School not attended<br>2) Primary school (Grade 7 or below)<br>3) High school (not completed)<br>4) Matric<br>5)<br>Post-matric (diploma, bachelor’s degree, post-graduate degree)

What is your current employment status?	1) Permanently employed<br>2) Employed part-time<br>3) Self-employed<br>4) Informally employed, piece jobs<br>5) Unemployed and looking for work<br>6) Unemployed and not looking for work<br>7)<br>Other (please specify)
Do you have a medical aid or hospital plan?	1) Yes<br>2) No<br>1) Other (please specify)
Do you or someone in your household currently receive a social grant?	1) Yes<br>2) No<br>If Yes, which of the following social grants do you and/or a family member receive?<br>1) Child support grant<br>2) Foster child grant<br>3) Old Age Pension<br>4) Disability<br>5) COVID-19 R350 Social Relief Grant<br>6) Other (please specify)

Choose the options that best describe the housing that you currently live in.

Material of the house	1) Concrete or wood<br>2) Mud or thatch
Roof material	1) Tiles or galvanized iron or concrete<br>2) Mud or thatch or plastic
Type of lighting	1) Electricity or gas<br>2) Candle or wood
Source of water	1) Piped into dwelling or borehole with pump or protected dug well<br>2) Pond or unprotected well
Number of people sharing a room in the house	1) 5 or fewer people per room<br>2) 6 or more people per room
Toilet facilities	1) Flush or ventilated improved latrine<br>2) Open pit or none (bush field)

Which of the following do you have in your household?

Sewing machine
Radio
TV
Stove
Fridge
Mobile phone
Bicycle
Motorbike
Car
Computer

Section 3: Health information	
Do have any of these health conditions?	1) Asthma/Chronic Obstructive Pulmonary Disease<br>2) Hypertension<br>3) HIV<br>4) Diabetes<br>5) Chronic Heart Disease<br>6) Chronic Kidney Disease<br>7) Cancer<br>8) Tuberculosis<br>9) Obesity<br>10) Other (please specify)<br>11) No, I don't have any existing health conditions

Section 4: Vaccinations	
Have you been vaccinated today?	1) Yes<br>2) No<br><br>Did a doctor or nurse here at the clinic tell you about the flu vaccine today?<br><br>1) Yes<br>2) No<br>3) Other (please specify)
Do you have any worries or concerns about the flu vaccine?	1) Yes, major concerns<br>Please specify<br>2) Yes, minor concerns<br>Please specify<br>3) No concerns<br>4) I don't know
What is the reason you chose not to get vaccinated?	1) I did not think it is necessary<br>2) I'm afraid of the immediate side effects<br>3) I don't trust vaccines<br>4) I don't worry about getting sick from the flu<br>5) Other (please specify)
What is the MAIN reason you chose not to get vaccinated?	1) I did not think it is necessary<br>2) I'm afraid of the immediate side effects<br>3) I don't trust vaccines<br>4) I don't worry about getting sick from the flu<br>5) Other (please specify)
Please indicate when you remember receiving flu vaccinations.	1) 2023 (this year)<br>2) 2022<br>3) 2021<br>4) 2020<br>5) 2019<br>6) 2018<br>7) Before 2018

	8) I have had flu vaccinations, but can't remember when<br>9) I have never received a flu vaccine
In the past, where have you received your flu vaccines?	1) Local clinic<br>2) Public hospital<br>3) Private Doctor<br>4) Private hospital<br>5) Pharmacy<br>6) Place of work<br>7) Other (please specify)

Section 5: Exposure to flu vaccination communications	
Have you seen or heard adverts about flu and/or flu vaccines recently (this year)?	1) Yes<br>2) No<br><br>If Yes, please indicate where:<br>1. In newspapers<br>2. On billboards or outdoors<br>3. On TV<br>4. On the radio<br>5. Online (websites, adverts)<br>6. On social media channels (this question refers to adverts, not personal posts)<br>7. In email newsletters from companies or organisations<br>8. Other (please specify)
What advertising about flu vaccines have you seen or heard?	1) Advertising by pharmacies (such as Dischem or Clicks)<br>2) Advertising by medical aids<br>3) Department of Health advertising or announcements<br>4) School or workplace messages<br>5) The Bambisana campaign<br>6) Other (please specify)
Please select all that apply:	1) I saw/was part of a flu vaccine community event.<br><br>2) I heard about a flu vaccine campaign, advert or event from someone I know and trust.<br><br>Please tell us what they said about the campaign or event.<br><br>3) A family member/friend advised me to get the flu vaccine.<br>4) A doctor/nurse advised me to get the flu vaccine.

	5) My religious leader advised me to get the flu vaccine.<br>6) A community leader/local organisation advised me to get the flu vaccine.<br>7) People in my community advised me to get the flu vaccine.<br>8) My employer advised me to get the flu vaccine.<br>9) My school/college/university advised me to get the flu vaccine.<br>10) I had always planned to get vaccinated.<br>1) Other (please specify)

Section 6: Potential barriers and risk perception

How easy or difficult is it to find a site to get vaccinated for the flu?	1) Very easy<br>2) Easy<br>3) Difficult<br>4) Very difficult
How easy or difficult is it to find transport to the site?	1) Very easy<br>2) Easy<br>3) Difficult<br>4) Very difficult
How easy or difficult is it to afford transport to the site?	1) Very easy<br>2) Easy<br>3) Difficult<br>4) Very difficult
How easy or difficult is it to find a vaccination site with convenient opening hours?	1) Very easy<br>2) Easy<br>3) Difficult<br>4) Very difficult

Did you have to take time off from work/school to come to the clinic?	1) Yes<br>2) No<br>3) Not applicable<br>If Yes, how easy or difficult is it to ge time off from work/school to come to the clinic?<br>1) Very easy<br>2) Easy<br>3) Difficult<br>4) Very difficult
Did you have to arrange childcare to come to the clinic?	1) Yes<br>2) No<br>3) Not applicable<br>If Yes, how easy or difficult is it to arrange childcare to come to the clinic?<br>1) Very easy<br>2) Easy

	3) Difficult<br>4) Very difficult
I don't worry about getting sick from cold or the flu	1) Strongly agree<br>2) Agree<br>3) Neutral<br>4) Disagree<br>5) Strongly disagree
Flu is seasonal; I will get better on my own	1) Strongly agree<br>2) Agree<br>3) Neutral<br>4) Disagree<br>5) Strongly disagree

Section 7: Attitudes

Indicate to what extent you agree or disagree with the following statements

"Immunisations are important for children to have"	1) Strongly agree<br>2) Agree<br>3) Neutral<br>4) Disagree<br>5) Strongly disagree
"Immunisations are safe"	1) Strongly agree<br>2) Agree<br>3) Neutral<br>4) Disagree<br>5) Strongly disagree
"Immunisations are effective"	1) Strongly agree<br>2) Agree<br>3) Neutral<br>4) Disagree<br>5) Strongly disagree
"Immunisations are compatible with my religious, personal and philosophical beliefs"	1) Strongly agree<br>2) Agree<br>3) Neutral<br>4) Disagree<br>5) Strongly disagree

Section 8: Internet access and use	
Do you have access to the internet?	1) Yes<br>2) No<br>If No, what is the reason? (select all that apply)<br>1) I don't own a device that can access the internet<br>2) My cell phone is not a smartphone<br>3) I can't afford data bundles or Wi-Fi<br>4) Other (please specify)<br>If Yes, which device or devices do you use to access the internet? (select all that apply)<br>1) Smartphone

	2) Tablet<br>3) Laptop<br>4) Other (please specify)
Where do you access the internet?	1) At home<br>2) At work, for work use only<br>3) At work, for work and/or personal use<br>4) From an internet cafe or public location such as Postnet<br>5) While travelling in a taxi/bus Other (please specify)
How do you connect to the internet?	1) Cell phone contract data package<br>2) General data bundles<br>3) WhatsApp-only data bundles<br>4) Facebook-only data bundles<br>5) Wi-Fi at home<br>6) Public Wi-Fi<br>7) Other (please specify)
Who usually pays for your data?	1) Myself<br>2) Parent<br>3) Boyfriend/girlfriend/partner<br>4) Sibling<br>Other (please specify)
Which social media platforms have you accessed in the past 30 days?	1) Facebook<br>2) Instagram<br>3) TikTok<br>4) Twitter<br>5) YouTube<br>6) Other (please specify)
Which social media platform do you use the most?	1) Facebook<br>2) Instagram<br>3) TikTok<br>4) Twitter<br>5) YouTube<br>6) Other (please specify)
Do you use WhatsApp?	1) Yes<br>2) No<br>If Yes, how do you use WhatsApp?<br>1) Communicating with individuals family, friends or colleagues<br>2) Participating in small WhatsApp groups (under 100 members)<br>3) Participating in large WhatsApp groups (over 100 members)<br>4) Broadcasting messages to multiple contacts (eg. for business)<br>If No, how do you communicate with people or groups?<br>1) SMS

	2) Telephone<br>3) Other platforms (please specify)
Are you a member of any WhatsApp groups?	1) Yes<br>2) No<br>If Yes, what do you use WhatsApp groups for? (select all that apply)<br>1) Messaging friends and family<br>2) Sharing news and content<br>3) Finding employment opportunities<br>4) Neighbourhood watch<br>5) Assessing and sharing health information<br>6) Other (please specify)
On average, how many hours do you spend online? (This excludes WhatsApp)	1) 1-2 hours<br>2) 2-4 hours<br>3) 4-6 hours<br>4) 6-8 hours<br>5) More than 8 hours
During which time of the day?	1) 6 am-12pm<br>2) 12pm-6pm<br>3) 6pm-10pm<br>4) After 10pm

Section 9: Sources of information and influence

Choose the top three: Which platforms do you think provide reliable, trustworthy information…

… about general health?	… about immunisations?
1) Television<br>2) Commercial radio (e.g. Metro FM, YFM, Kaya FM)<br>3) Community radio (e.g. Jozi FM, Alex FM)<br>4) National newspapers<br>5) Local newspapers<br>6) Government communication (e.g. website, SMS)<br>7) Social media (e.g. Facebook, TikTok, Instagram)<br>8) The Internet (e.g. Google)<br>9) WhatsApp<br>10) Other (please specify)	1) Television<br>2) Commercial radio (e.g. Metro FM, YFM, Kaya FM)<br>3) Community radio (e.g. Jozi FM, Alex FM)<br>4) National newspapers<br>5) Local newspapers<br>6) Government communication (e.g. website, SMS)<br>7) Social media (e.g. Facebook, TikTok, Instagram)<br>8) The Internet (e.g. Google)<br>9) WhatsApp<br>10) Other (please specify)

Select the top three that apply: Which of the following social media and messaging platforms do you think provide reliable, trustworthy information …

… about general health?	… about vaccines?
1) Facebook<br>2) Instagram<br>3) Twitter<br>4) TikTok<br>5) YouTube<br>6) WhatsApp<br>7) Other (please specify)	1) Facebook<br>2) Instagram<br>3) Twitter<br>4) TikTok<br>5) YouTube<br>6) WhatsApp<br>7) Other (please specify)

Select the top three people that you would go to for reliable, trustworthy information …

… about general health?	… about immunisations?
1) Family/friends<br>2) Influential community members<br>3) Traditional healers<br>4) NGOs or community support organisations<br>5) Religious leaders<br>6) Local clinic nurse<br>7) Local clinic doctor<br>8) Social media influencers<br>9) My employer and colleagues<br>10) Scientists and other experts<br>11) Government and politicians<br>12) Other (please specify)	1) Family/friends<br>2) Influential community members<br>3) Traditional healers<br>4) NGOs or community support organisations<br>5) Religious leaders<br>6) Local clinic nurse<br>7) Local clinic doctor<br>8) Social media influencers<br>9) My employer and colleagues<br>10) Scientists and other experts<br>11) Government and politicians<br>12) Other (please specify)

Section 10: Knowledge of flu vaccination	
How often should people get vaccinated against flu?	1) Every year<br>2) Once<br>3) Never<br>4) Don’t know<br>5) Other (please specify)
Who should get vaccinated against flu?	1) Everyone<br>2) Adults 65 and older<br>3) Pregnant women<br>4) People with chronic disease<br>5) Healthcare professionals

During which time of the year do people in South Africa get sick from flu?	1) All year<br>2) During winter months<br>3) During the summer months<br>4) Other (please)
Indicate to what extent you agree or disagree with the following statements	

"Being vaccinated reduces the severity and duration of flu"	1) Strongly agree<br>2) Agree<br>3) Neutral<br>4) Disagree<br>5) Strongly disagree
"Being vaccinated against flu improves immunity"	1) Strongly agree<br>2) Agree<br>3) Neutral<br>4) Disagree<br>5) Strongly disagree
"Severe illness and complication from flu can lead to absence from school or work and affect quality of work."	1) Strongly agree<br>2) Agree<br>3) Neutral<br>4) Disagree<br>5) Strongly disagree
"Severe illness from flu can lead to hospitalisation or even death."	1) Strongly agree<br>2) Agree<br>3) Neutral<br>4) Disagree<br>5) Strongly disagree
"Flu vaccination is effective if someone is already infected with flu"	1) Strongly agree<br>2) Agree<br>3) Neutral<br>4) Disagree<br>5) Strongly disagree

You have reached the end of the survey.
