# Supplementary material for: Assessing Community and Social Media Influence to Increase Influenza Vaccine Uptake among Youth in Soweto, South Africa (The Bambisana Study): Protocol for a Mixed Methods Pretest-Posttest Intervention Study
Source: JMIR Res Protoc. 2025 Jun 17;14:e60481. doi: 10.2196/60481 (PMC12214695; doi:10.2196/60481)
Supplement: Multimedia Appendix 5 [file resprot_v14i1e60481_app5.docx]

**Focus Group Discussions/Key informant interview: Topic Guide_ Young people and NDoH Priority Group participants**

Assessing community and social media influence: motivating influenza vaccination among youth.

*Qualitative research objective:* To collect contextual data that could be used to explain the **motivators, barriers, and key important influencers** that affect young people when it comes to influenza vaccination decision-making.

# Introduction, and demographics

Good day, my name is………………………………and my colleague is………………………….. We are

working at the Wits VIDA, a Wits University research unit and a division of the Wits Health Consortium based at Chris Hani Baragwanath Hospital. Thank you for agreeing to take part in this interview.

Before we switch on the recorder, please remember there is no right or wrong answer. Your time and experiences are valuable, and we want you to feel respected and comfortable. Now I’m going to turn on the audio recorder, is that OK?” Wait to hear a verbal yes

# General vaccine attitudes (10 mins)

1. I’d like to start by talking a bit about the influenza vaccines. Let’s start with a general question
2. Do you know what the influenza vaccine is? /what is it used for?
3. What words come to mind when you think of the influenza vaccines?
4. How does the idea of an influenza vaccine make you feel?
5. Have you can been vaccinated?

If yes, What motivated you?

If no, why did you decide not to get vaccinated?

# Credible voices and communication (30 mins)

I’d like to start by talking a bit about what sources of information you trust and use to access information.

1. Please share what your trusted sources of information are to access information?
   1. What media have you engaged with in the last month?
   2. What media do you trust the most? Why do you trust these sources?
   3. What media do you trust the least? Why do you not trust these sources?

1. I’m going to go around the room and ask you each to say a word or phrase that best sums up your attitude towards the media in South Africa right now.
2. Hands up if you have access to a smartphone or device that allows you to use social media? *Facilitator to count how many have access to a smartphone device and give the number oud loud for the audio recorder to capture.* a. What do you tend to use social media for?

1. What general health information have you searched online and what platform did you use? Probes: Social media, google, other sources and also probe for any vaccine related information.

1. For who do you think is social media influential on topics related to health and vaccines (young vs. old people)?
   1. Which specific platforms are more influential sources?
   2. What types of content are more influential? PROBE: **Type:** Text, Images,

Videos**; Content:** Instructional / Informational, News, Opinions / Opinion pieces, Real world (e.g. people taking the vaccines), **Origin:** Shared by friends or family / celebrities or influencers / users in groups or communities / shared by unknown users / Promoted content

1. For who is social media LESS influential on topics related to health and vaccines?
2. What other types of platforms /sources of information are more influential instead for these audiences? Probes: **Type:** TV, Traditional media, radio, community

(offline) influencers. **Content:** News articles, flyers, TV and radio adverts, events; **Origin:** Shared by healthcare providers, influencial people in the community, friends and family etc

1. What type of content are more influential for these audiences?

# Access and exposure to the campaign and flu vaccine ads (30 mins)

Now I’d like to talk about other sources that you rely on for information about vaccines.

1. Which people or groups of people have been most influential in shaping your views about vaccines in general or the flu vaccine specifically?

*Probes: Types of infuencers to be potentially discussed:*

- 1. Healthcare professionals
  2. Politicians (provincial and national government)
  3. Friends and family
  4. Influencal people in your community/community leaders, e.g. councillors, CAB members, religious or tribal leaders, local sportspeople, artists, etc
  5. Social media influencers and celebrities
  6. National public health authorities, e.g. NICD
  7. International health authorities, e.g. Africa CDC

1. Why do you trust their opinions?
2. Have any of these people influenced your opinions or decision-making surrounding vaccination? How?

1. Have you ever received communications from these people on the topic of health and vaccination? If yes, what did these communications say?

Now I would like to ask you about your exposure to vaccine communications more generally.

1. Thinking specifically about traditional media (TV, radio etc) and offline sources of information (community campaign, flyers). What, if anything, have you seen this flu season about influenza vaccines?
   - 1. Which platforms come to mind? E.g. news articles, radio, TV, email marketing, flyers, events, etc
     2. What campaigns or content come to mind?
     3. Who delivered this campaign/content?

1. Do you remember seeing any posters, flyers or events by **the Bambisana** campaign? *Moderator to make clear what this campaign is and if they don’t know, to show them all the campaign materials – online and offline.*

If yes:

- - 1. How did you feel about this content/these event?
    2. How accurate did you think this information they provided was?
    3. What new or suprising information did you learn from this content? Did you find it helpful? If yes, why?

1. Now thinking specifically about social media. What, if any, information have you seen this flu season on social media about influenza vaccines?
   - 1. Which platforms come to mind? E.g. Twitter, Facebook, TikTok, etc
     2. What campaigns or content come to mind?
     3. Who delivered this campaign/content?

1. Do you remember seeing any social media content from the Bambisana campaign?

If yes:

- - 1. How did you feel about this content?
    2. How accurate did you think this information they provided was?
    3. What new or suprising information did you learn from this content? Did you find it helpful? If yes, why?

1. For the Bambisana campaign, community influencers shared information about the campaign and general information about the flu vaccine.
   - 1. Via which channels did you receive this information? Probes: word of mouth,

WhatsApp

- - 1. What information this you receive?
    2. What were your thoughts on this information?

# Creative testing (30 mins)

MODERATOR READ: I’m now going to show you a selection of content featuring different messages. I want you to pay close attention to it and then we are going to discuss some elements of it afterwards. Please understand that I did not make this content and that I am an independent researcher. I want to see your reactions to the content whether you like it or dislike it. We value your feedback and honest opinion as it wil help us to improve the content.

The moderator pass round Bambisana flyers (offline) and then show one of the Bambisana campaign films (social media, projected in a screen)

*For each piece of content shown, ask:*

1. What is the main message of this content?
2. What parts are most impactful?
3. What – if anything - did you like about the way that the message was communicated?
4. Was there anything you didn’t like?
5. Did the content change your opinion about flu vaccination, or teach you anything you didn’t previously know about it?
6. After seeing this content, how likely do you think it is that it would change the opinion of peers/young people in your community on receiving the influenza vaccination?
7. How likely would you be to like or share a piece of content like this if you saw it online? Why?

***Moderator to share information below with the participants***

# Difference between Vaccination and Immunisation

The terms ‘vaccination’ and ‘immunisation’ are similar, but don’t exactly mean the same thing. Vaccination is the term used for getting a vaccine — that is, actually having the injection or taking an oral vaccine dose. Immunisation is the process of both getting the vaccine and becoming immune to the disease after vaccination.
