# Supplementary material for: Assessing Community and Social Media Influence to Increase Influenza Vaccine Uptake among Youth in Soweto, South Africa (The Bambisana Study): Protocol for a Mixed Methods Pretest-Posttest Intervention Study
Source: JMIR Res Protoc. 2025 Jun 17;14:e60481. doi: 10.2196/60481 (PMC12214695; doi:10.2196/60481)
Supplement: Multimedia Appendix 6 [file resprot_v14i1e60481_app6.docx]

**KII Topic Guide- Community Leaders/Influencers**

Assessing community and social media influence: motivating influenza vaccination among youth.

*Qualitative research objective:* To collect contextual data that could be used to explain the **motivators, barriers, and key important influencers** that affect young people when it comes to influenza vaccination decision-making.

1. **Introduction**

Good day, my name is………………………………and my colleague is………………………….. We are working at the Wits VIDA, a Wits University research unit and a division of the Wits Health Consortium based at Chris Hani Baragwanath Hospital. Thank you for agreeing to take part in this interview.

We have asked you to participate because you interact with young people aged 18-34 who live (study clusters).We would like for you to share your experiences in working/interacting with young people aged 18-34 years and vaccines campaigns

Before we switch on the recorder, please remember there is no right or wrong answer. Your time and experiences are valuable, and we want you to feel respected and comfortable. Now I’m going to turn on the audio recorder, is that OK?” Wait to hear a verbal yes.

**i. Community Leader/influencer profiling**

- Can you tell me about your role as a community influencer? Probes: What kind of influence do you have? Who or what inspired you to become a community leader/influencer?
- Who are the people you target/engage with in your work? Probes: describe their characteristics (Age, Gender, Community, Employment), how many do you engage with on a daily, weekly, or monthly basis?
- How do you reach/engage with people? Probes: Online, community (where, which places?), community meetings/events, place of work etc
- Can you talk me through your approach to community engagement? Probes: strategies used to help guide and support the community
- What are the types of events do you usually participate in / run as a community leader/influencer? Probes: network (what other organizations/stakeholders do you engage/work with?
- Do people seek support from you on health related matter? If, so what kinds of matters? Probing: mental health support, family planning, vaccine hesitancy

1. **General vaccine attitudes (10 mins)**
2. I’d like to start by talking a bit about the influenza vaccines. Let’s start with some general questions
   1. Do you know what the influenza vaccine is? What is it used for? How does it work?
   2. What words come to mind when you think of the influenza vaccines?
   3. How does the idea of being vaccinated for influenza make you feel?
   4. Are there certain types of vaccines you feel more confident about taking? If so, which ones? And why?
3. Influenza vaccine

- Have you been vaccinated?
- If yes, what motivated you?
- If no, why did you decide not to get vaccinated?

1. Community vaccine attitudes

- What are the vaccine attitudes in your community?
- What are some of the barriers towards/concerns around influenza vaccination?
- What are some of the rumours or misconceptions about vaccines from your community?
- What are some of the facilitators for influenza vaccination?
- What are the main sources of information for community members about influenza vaccines?

**For Healthcare workers**

- What activities do you have in place during the influenza vaccination period to promote influenza vaccination?
- Can you share your experience in administering the influenza vaccine?
- Do you routinely offer influenza vaccination to all patients who come to the healthcare centre during the vaccination season?
- What are the challenges or barriers you and your colleagues experience in the routine provision of the influenza vaccine? (*Probe:* healthcare setting problems, patient behaviour, perceptions, resources)
- What do you think can be done to promote the uptake of the influenza vaccines?

1. **Access and exposure to the campaign and flu vaccine ads (30 mins)**

I would like to ask you about your exposure to vaccine communications more generally.

1. What campaign, if any, have you seen this flu season about influenza vaccines? (Probing: Government related campaigns, the Bambisana Campaign)
   1. Which platforms come to mind? PROBING: Traditional media (TV, radio etc) and offline sources of information (community campaign, flyers); SOCIAL MEDIA:Twitter, FB, Tiktok, WhatsApp etc.
   2. Who delivered this campaign/content?
   3. What campaign content comes to mind of the ones you have talked about? (Probing: Informational, educational, type of vaccines, vaccination site information etc.)
   4. How did you feel about this content/these events?
   5. How accurate did you think this information they provided was?
   6. What new or suprising information did you learn from this content? Did you find it helpful? If yes, why?

**For Bambisana community influencers only:**

1. For the Bambisana campaign, you were a community influencer that shared information about the campaign and general information about the flu vaccine.
   1. Via which channels did you share this information? Probes: word of mouth, WhatsApp
   2. What information did you share?
   3. What type of audience did you reach? Probes: Describe their characteristics, age, gender, community etc.
   4. What feedback did you receive from your audience about the information that you shared?
   5. What worked well performing your role as a community influencer?
   6. What were the barriers/challenges during this role?
2. **Creative testing (30 mins)**

MODERATOR READ: I’m now going to show you a selection of content featuring different messages. I want you to pay close attention to it and then we are going to discuss some elements of it afterwards. Please understand that I did not make this content and that I am an independent researcher. I want to see your reactions to the content whether you like it or dislike it. We value your feedback and honest opinion as it wil help us to improve the content.

Moderator pass round Bambisana flyers (offline) and then show one of the Bambisana campaign films (social media, projected in a screen)

*For each piece of content shown, ask:*

1. What is the main message of this content?
2. What parts are most impactful?
3. What – if anything - did you like about the way that the message was communicated?
4. Was there anything you didn’t like?
5. Did the content change your opinion about flu vaccination, or teach you anything you didn’t previously know about it?
6. After seeing this content, how likely do you think it is that it would change the opinion of peers/young people in your community on receiving the influenza vaccination?
7. How likely would you be to like or share a piece of content like this if you saw it online? Why?

***Moderator to share information below with the participants (if required)***

Difference between Vaccination and Immunisation

The terms ‘vaccination’ and ‘immunisation’ are similar, but don’t exactly mean the same thing. Vaccination is the term used for getting a vaccine — that is, actually having the injection or taking an oral vaccine dose. Immunisation is the process of both getting the vaccine and becoming immune to the disease after vaccination.
